# ACE2 activator diminazene aceturate ameliorates Alzheimer's disease-like neuropathology and rescues cognitive impairment in SAMP8 mice

**DOI:** 10.18632/aging.103544

**Published:** 2020-07-23

**Authors:** Rui Duan, Xiao Xue, Qiao-Quan Zhang, Si-Yu Wang, Peng-Yu Gong, Yan E, Teng Jiang, Ying-Dong Zhang

**Affiliations:** 1 School of Basic Medicine and Clinical Pharmacy, China Pharmaceutical University, Nanjing 211198, People's Republic of China; 2 Department of Neurology, Nanjing First Hospital, Nanjing Medical University, Nanjing 210006, People's Republic of China; 3 Department of Pathology, Nanjing Brain Hospital, Nanjing Medical University, Nanjing 210029, People's Republic of China

**Keywords:** Alzheimer’s disease, ACE2, diminazene aceturate, Ang-(1-7)

## Abstract

Previously, we revealed that brain Ang-(1-7) deficiency was involved in the pathogenesis of sporadic Alzheimer’s disease (AD). We speculated that restoration of brain Ang-(1-7) levels might have a therapeutic effect against AD. However, the relatively short duration of biological effect limited the application of Ang-(1-7) in animal experiments. Since Ang-(1-7) is generated by its metabolic enzyme ACE2, we then tested the efficacy of an ACE2 activator diminazene aceturate (DIZE) on AD-like neuropathology and cognitive impairment in senescence-accelerated mouse prone substrain 8 (SAMP8) mice, an animal model of sporadic AD. Eight-month-old SAMP8 mice were injected intraperitoneally with vehicle or DIZE once a day for 30 consecutive days. DIZE markedly elevated brain Ang-(1-7) and MAS1 levels. Meanwhile, DIZE significantly reduced the levels of Aβ_1-42_, hyperphosphorylated tau and pro-inflammatory cytokines in the brain. The synaptic and neuronal losses in the brain were ameliorated by DIZE. Importantly, DIZE improved spatial cognitive functions in the Morris water maze test. In conclusion, this study demonstrates that DIZE ameliorates AD-like neuropathology and rescues cognitive impairment in SAMP8 mice. These beneficial effects of DIZE may be achieved by activating brain ACE2/Ang-(1-7)/MAS1 axis. These findings highlight brain ACE2/Ang-(1-7)/MAS1 axis as a potential target for the treatment of sporadic AD.

## INTRODUCTION

Alzheimer’s disease (AD) is an aging-related neurodegenerative disorder and the fourth leading cause of death in developed countries [1, 2]. Sporadic AD accounted for over 90% of all AD cases [3]. Pathologically, it is characterized by overproduction of amyloid-β (Aβ), hyperphosphorylation of tau and chronic inflammatory responses in the brain [4]. To date, the pathogenesis of sporadic AD remained largely unclear.

ACE2/Ang-(1-7)/MAS1 is a newly identified axis of renin-angiotensin system [5]. Our previous studies revealed that the levels of Ang-(1-7), the main effector of ACE2/Ang-(1-7)/MAS1 axis, were reduced in the plasma of sporadic AD patients as well as the brain tissues of AD animal models [6, 7]. Meanwhile, this reduction was closely correlated with progression of neuropathology and deterioration of cognitive functions [6, 7]. These findings implied that Ang-(1-7) deficiency contributed to the pathogenesis of sporadic AD. Based on these findings, we then speculated that restoration of brain Ang-(1-7) levels might have a therapeutic effect against AD progression.

However, as a heptapeptide, Ang-(1-7) possesses a relatively short duration of biological effect in vivo because it can be rapidly inactivated and degraded by several proteases [8, 9]. This property limited its direct application in animal experiments. Ang-(1-7) is mainly generated by its metabolic enzyme ACE2 [10, 11]. Emerging evidence suggested that diminazene aceturate (DIZE), a classic ACE2 activator, was able to steadily increase brain Ang-(1-7) levels and thus exerted beneficial effects in animal models of ischemic stroke, anxiety disorder and dementia [12–15]. In light of this evidence, we tried to test the efficacy of DIZE on AD-like neuropathology and cognitive impairment in senescence-accelerated mouse prone substrain 8 (SAMP8) mice, an animal model of sporadic AD [16, 17], in this study.

## RESULTS

### DIZE activated ACE2/Ang-(1-7)/MAS1 axis in the brain of SAMP8 mice

**Figure 1:**
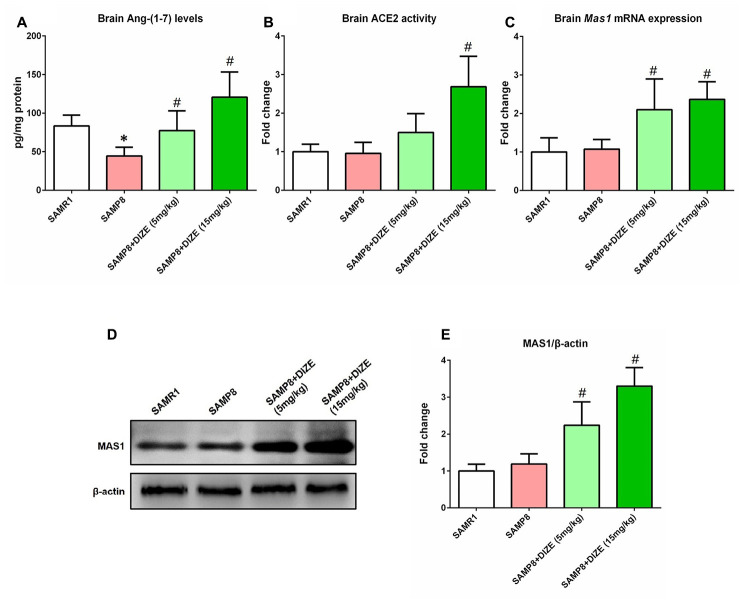
**DIZE activated ACE2/Ang-(1-7)/MAS1 axis in the brain of SAMP8 mice.** (**A**) The Ang- (1-7) levels in mice brain were detected by ELISA. (**B**) The activity of ACE2 in mice brain was assessed using a specific detection kit (#AS-72086, AnaSpec, Inc., Fremont, CA, USA) with Mc-Ala/Dnp fluorescence resonance energy transfer peptides as described. The fluorescence of Mc-Ala was monitored at excitation/emission 330 nm/390 nm. The specificity was confirmed using a specific ACE2 inhibitor DX600. (**C**) The *Mas1* mRNA levels in mice brain were evaluated by qRT-PCR, and Gapdh was used as an internal control. (**D**) The protein levels of MAS1 in mice brain were detected by western blot. β-actin was used as a loading control. (**E**) Quantitative analysis of MAS1 protein levels. Data from panel B, C and E were expressed as a fold change relative to the vehicle-treated age-matched SAMR1 control mice. All data were analyzed by one-way ANOVA followed by Tukey’s post hoc test. Columns represent mean ± SD (n=8 per group). *P<0.05 versus age-matched vehicle-treated SAMR1 control mice. #*P*<0.05 versus vehicle-treated SAMP8 mice.

As revealed by Figure 1A, the Ang-(1-7) levels in the brain of SAMP8 mice were significantly lower than those of senescence-accelerated mouse resistant substrain 1 (SAMR1) mice (44.49 pg/mg vs. 83.30 pg/mg, *P*<0.05). Injection of DIZE (15 mg/kg) significantly increased brain ACE2 activity in SAMP8 mice (*P*<0.05, Figure 1B). Meanwhile, DIZE injection (5 mg/kg and 15 mg/kg) markedly elevated Ang-(1-7) levels in the brain of SAMP8 mice (5 mg/kg DIZE: 77.41 pg/mg vs. 44.49 pg/mg; 15 mg/kg DIZE: 120.60 pg/mg vs. 44.49 pg/mg; all *P*<0.05, Figure 1A). The *Mas1* mRNA expression in the brain of SAMP8 mice was increased following DIZE injection (5 mg/kg and 15 mg/kg, *P*<0.05, Figure 1C), and this alteration was confirmed by western blot analysis at the protein level (5 mg/kg and 15 mg/kg, *P*<0.05, Figure 1D and 1E).

### DIZE reduced Aβ_1-42_ levels in the brain of SAMP8 mice

**Figure 2:**
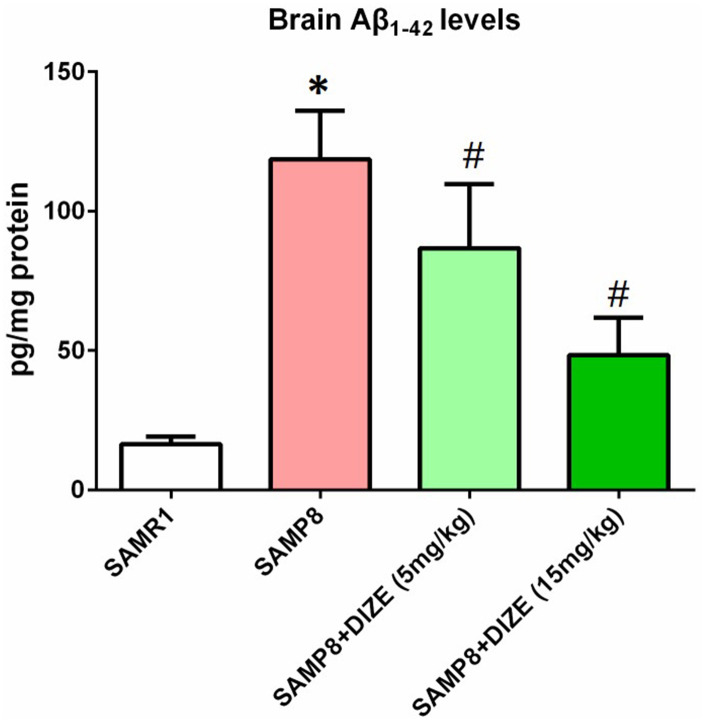
**DIZE reduced Aβ_1-42_ levels in the brain of SAMP8 mice.** The levels of TBS-soluble Aβ_1-42_ in the brain were detected by ELISA. Data were analyzed by one-way ANOVA followed by Tukey’s post hoc test. Columns represent mean ± SD (n=8 per group). *P<0.05 versus age-matched vehicle-treated SAMR1 control mice. #*P*<0.05 versus vehicle-treated SAMP8 mice.

As indicated by Figure 2, SAMP8 mice showed higher brain Aβ_1-42_ levels when compared with their age-matched SAMR1 control mice (*P*<0.05). DIZE injection significantly reduced Aβ_1-42_ levels in the brain of SAMP8 mice (5 mg/kg and 15 mg/kg, *P*<0.05, Figure 2). DIZE injection (15 mg/kg) had no effect on Aβ_1-42_ levels in the brain of SAMR1 control mice (data not shown).

### DIZE ameliorated tau hyperphosphorylation in the brain of SAMP8 mice

**Figure 3:**
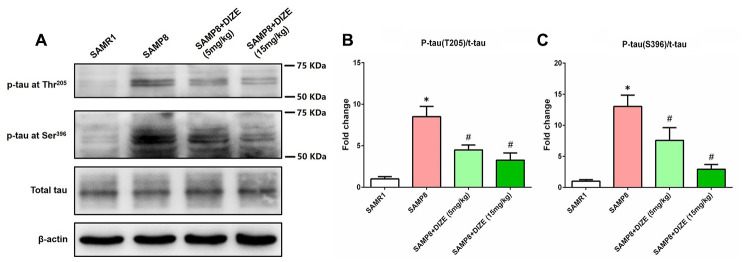
**DIZE ameliorated tau hyperphosphorylation in the brain of SAMP8 mice.** (**A**) The levels of tau hyperphosphorylation at Thr205 and Ser396 sites as well as total tau in the brain were detected by western blot. β-actin was used as a loading control. (**B**) Quantitative analysis of tau hyperphosphorylation at Thr205/total tau ratio. (**C**) Quantitative analysis of tau hyperphosphorylation at Ser396/total tau ratio. Data from panel B and C were expressed as a fold change relative to the age-matched vehicle-treated SAMR1 control mice. Data were analyzed by one-way ANOVA followed by Tukey’s post hoc test. Columns represent mean ± SD (n=8 per group). *P<0.05 versus age-matched vehicle-treated SAMR1 control mice. #*P*<0.05 versus vehicle-treated SAMP8 mice.

In the brain of SAMP8 mice, tau hyperphosphorylation at Thr205 (Figure 3A and 3B) and Ser396 (Figure 3A and 3C) sites was noted (*P*<0.05). As indicated by Figure 3A and 3B, injection of DIZE (5 mg/kg and 15 mg/kg) significantly attenuated tau hyperphosphorylation at Thr205 site (*P*<0.05). Meanwhile, levels of hyperphosphorylated tau at Ser396 site in the brain of SAMP8 mice were also decreased by DIZE injection (5 mg/kg and 15 mg/kg, *P*<0.05, Figure 3A and 3C). DIZE injection (15 mg/kg) had no effect on hyperphosphorylated tau levels in the brain of SAMR1 control mice (data not shown).

### DIZE attenuated neuroinflammation in the brain of SAMP8 mice

**Figure 4:**
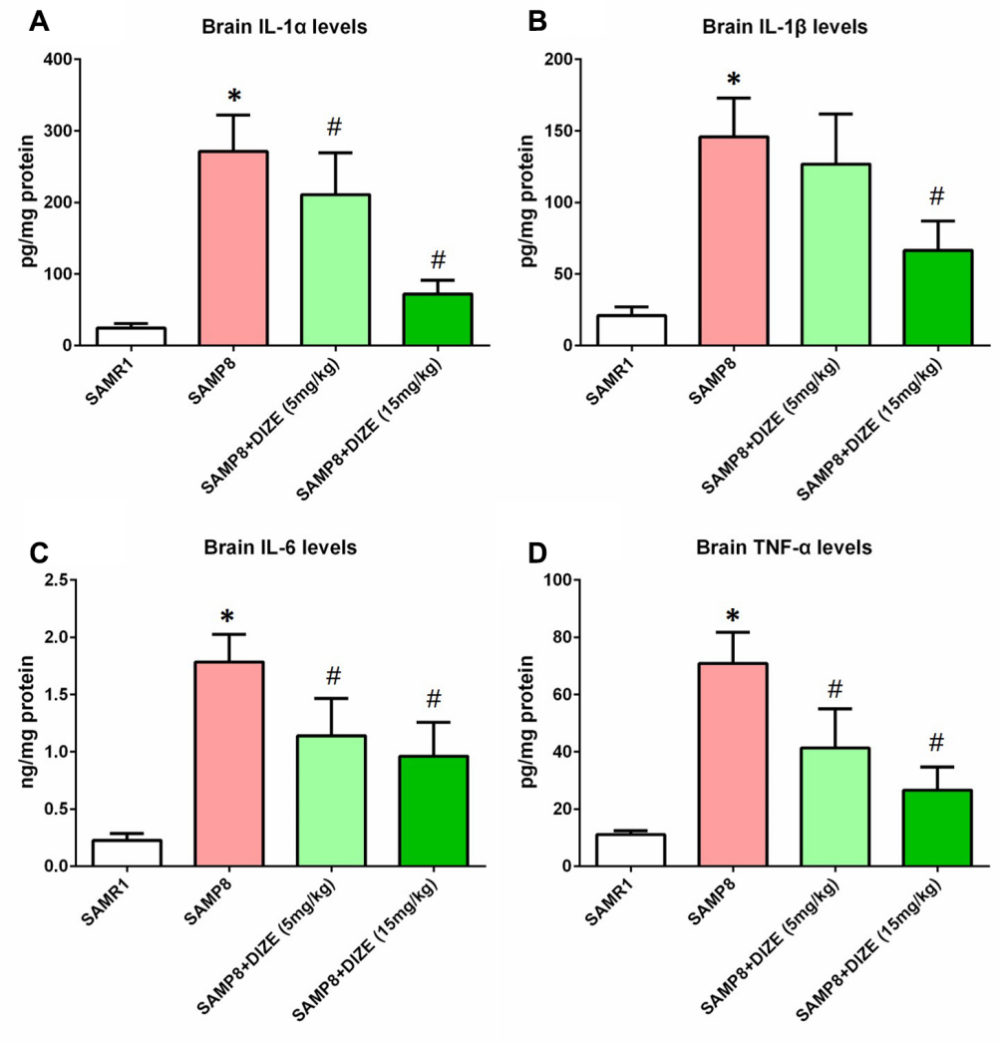
**DIZE attenuated neuroinflammation in the brain of SAMP8 mice.** (**A**) The protein levels of IL-1α in the brain were investigated by ELISA. (**B**) The protein levels of IL-1β in the brain were investigated by ELISA. (**C**) The protein levels of IL-6 in the brain were investigated by ELISA. (**D**) The protein levels of TNF-α in the brain were investigated by ELISA. All data were analyzed by one-way ANOVA followed by Tukey’s post hoc test. Columns represent mean ± SD (n=8 per group). *P<0.05 versus age-matched vehicle-treated SAMR1 control mice. #*P*<0.05 versus vehicle-treated SAMP8 mice.

As indicated by Figure 4A–4D, the levels of pro-inflammatory cytokines including IL-1α, IL-1β, IL-6 and TNF-α in the brain of SAMP8 mice were significantly higher than those of SAMR1 control mice (*P*<0.05). As revealed by Figure 4A, 4C and 4D, DIZE injection (5 mg/kg and 15 mg/kg) markedly reduced the levels of IL-1α, IL-6 and TNF-α in the brains of SAMP8 mice (*P*<0.05). Meanwhile, brain IL-1β levels were significantly decreased by 15 mg/kg DIZE in SAMP8 mice (*P*<0.05, Figure 4B). Injection of DIZE (15 mg/kg) had no influence on the levels of IL-1α, IL-1β, IL-6 and TNF-α in the brain of SAMR1 control mice (data not shown).

### DIZE alleviated synaptic and neuronal losses in the brain of SAMP8 mice

**Figure 5:**
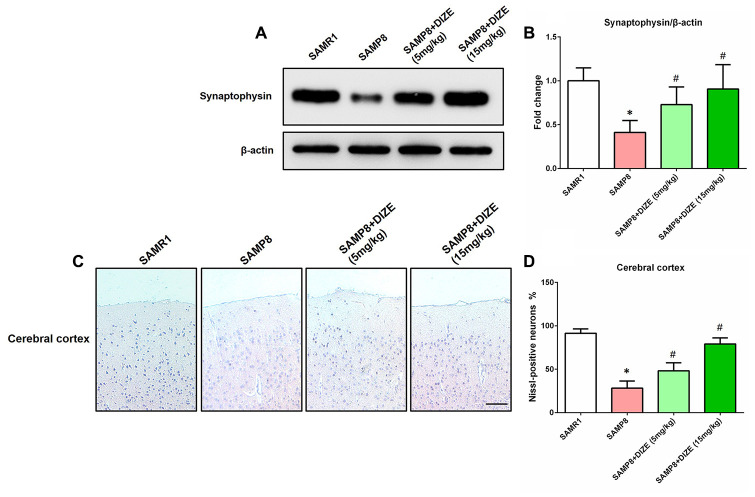
**DIZE alleviated synaptic and neuronal losses in the brain of SAMP8 mice.** (**A**) The protein levels of synaptophysin in the brain were detected by western blot. β-actin was used as a loading control. (**B**) Quantitative analysis of synaptophysin protein levels. Data were expressed as a fold change relative to the age-matched vehicle-treated SAMR1 control mice. (**C**) Neuronal loss in the parietal cortex of mice were detected by Nissl staining. Neurons with dark violet nucleus and intact morphology were identified as Nissl-positive neurons. Scale bar=100 μm. (**D**) Quantitative analysis of Nissl-positive neurons in the brain. Data were analyzed by one-way ANOVA followed by Tukey’s post hoc test. Columns represent mean ± SD (n=8 per group). *P<0.05 versus age-matched vehicle-treated SAMR1 control mice. #*P*<0.05 versus vehicle-treated SAMP8 mice.

As indicated by Figure 5A and 5B, SAMP8 mice exhibited apparent synaptic loss (indicated by protein levels of synaptophysin) when compared with their age-matched SAMR1 control mice (*P*<0.05). Meanwhile, an obvious neuronal loss (indicated by Nissl-positive neurons) in the parietal cortex was also noted in the brain of SAMP8 mice (Figure 5C and 5D, *P*<0.05). As revealed by Figure 5A and 5B, DIZE injection (5 mg/kg and 15 mg/kg) led to a significant increase in synaptophysin protein levels (*P*<0.05). Meanwhile, the percentage of Nissl-positive neurons in the parietal cortex was significantly increased after DIZE injection (Figure 5C and 5D, 5 mg/kg and 15 mg/kg, *P*<0.05). DIZE injection (15 mg/kg) had no influence on the synaptophysin protein levels or the percentage of Nissl-positive neurons in the brain of SAMR1 control mice (data not shown).

### DIZE rescued spatial cognitive impairment in SAMP8 mice

**Figure 6:**
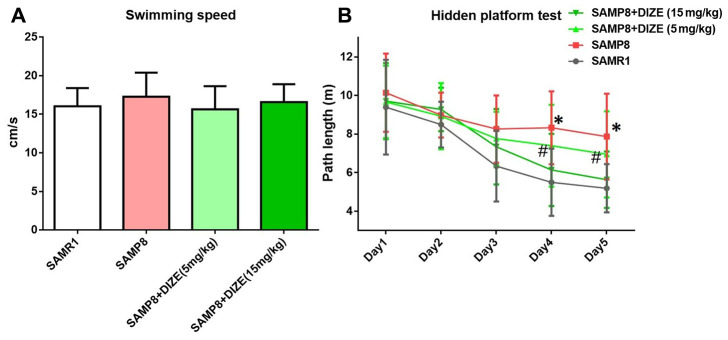
**DIZE rescued spatial cognitive impairment in SAMP8 mice.** (**A**) Swimming speed of each group in the MWM test. Data were analyzed by one-way ANOVA followed by Tukey’s post hoc test. (**B**) Path length of each group in the hidden platform task. Data were analyzed by two-way repeated measures ANOVA followed by Bonferroni’s multiple comparisons test. Columns represent mean ± SD (n=12 per group). *P<0.05 versus age-matched vehicle-treated SAMR1 control mice. #*P*<0.05 versus vehicle-treated SAMP8 mice.

Morris water maze (MWM) test was performed during the last 5 days before mice were killed. First, we compared the swimming speed between SAMP8 mice and their age-matched SAMR1 control mice, but no difference was noted (Figure 6A). DIZE injection did not significantly affect swimming speed in SAMP8 mice (5 mg/kg and 15 mg/kg, Figure 6B) or their age-matched SAMR1 control mice (15 mg/kg, data not shown). Afterwards, we employed a hidden platform test to assess the spatial cognitive functions in SAMP8 mice and their age-matched SAMR1 control mice. SAMP8 mice swam more distance than their age-matched SAMR1 control mice to find the hidden platform from day 4 to day 5 (day 4: 8.325±1.891 vs. 5.497±1.739 m; day 5: 7.869±2.226 vs. 5.188±1.25 m; n=12 per group, *P*<0.05). Two-way repeated measures ANOVA indicated that SAMP8 mice performed worse than their age-matched SAMR1 control mice during the whole task (*F*_genotype_ (1, 110)=27.82, *P*<0.05), confirming that SAMP8 mice displayed evident spatial cognitive impairment at 9 months of age. As indicated by Figure 6B, DIZE injection (15 mg/kg) rescued this spatial cognitive impairment in SAMP8 mice (*F*_treatment_ (1, 110)=11.41, *P*<0.05). DIZE injection (15 mg/kg) had no impact on spatial cognitive impairment in SAMR1 control mice (data not shown).

## DISCUSSION

In this study, we showed that the levels of Ang-(1-7), the main effector of ACE2/Ang-(1-7)/MAS1 axis, were reduced in the brain of SAMP8 mice. This was in accordance with our previous observations in AD animal models [7, 18]. These findings indicated that Ang-(1-7) deficiency might be involved in the progression of AD. Interestingly, in the brain of SAMP8 mice, we did not observe significant alteration in the activity of ACE2, the metabolic enzyme for Ang-(1-7) generation. Since Ang-(1-7) can be degraded by several proteases such as ACE and neutral endopeptidase *in vivo* [10, 11], we speculated that the reduction of Ang-(1-7) might be attributed to the accelerated proteolysis in the brain of SAMP8 mice.

Next, we tried to restore the levels of brain Ang-(1-7) using DIZE, a classic ACE2 activator. Previous findings indicated that DIZE might cross the blood–brain barrier and activated central ACE2 [14, 15]. In this study, we showed that DIZE significantly increased brain ACE2 activity and thus led to elevated Ang-(1-7) levels. Interestingly, an increased level of brain MAS1, the receptor for Ang-(1-7), was noted following DIZE treatment. This can be explained by the positive regulation of elevated Ang-(1-7) on its receptor MAS1, as previously reported by Xie and colleagues [19]. All these findings indicated that DIZE could activate brain ACE2/Ang-(1-7)/MAS1 axis.

Accumulation of Aβ within the brain represents a trigger of pathological cascades in AD [20]. In this study, we showed that DIZE diminished the levels of Aβ_1-42,_ the most toxic form of Aβ, in the brain of SAMP8 mice. ACE2 shares similar biofunctions to its homologue ACE [21], and recent evidence indicated that activation of ACE could reduce Aβ_1-42_ via converting it to a shorter Aβ form with less toxic [22]. Based on this evidence, we speculated that activation of ACE2 by DIZE decreased brain Aβ_1-42_ levels through a similar manner. This speculation needed to be verified by future studies.

In this study, we revealed that DIZE ameliorated tau hyperphosphorylation in the brain of SAMP8 mice. Since hyperphosphorylation of tau represents a downstream pathological hallmark triggered by Aβ_1-42_ [23], the reduction of hyperphosphorylated tau in this scenario might be a consequence of decreased Aβ_1-42_ levels caused by DIZE. In addition, DIZE also elevated Ang-(1-7) levels by activation of ACE2, while increased Ang-(1-7) could directly inhibit the activity of MAPK [24], an important kinase involved in hyperphosphorylating tau protein [25, 26]. This might represent another possible mechanism by which DIZE ameliorated tau hyperphosphorylation.

Chronic neuroinflammation was recently considered as another pathological hallmark of AD [27]. In this study, we showed that DIZE attenuated neuroinflammation in the brain of SAMP8 mice, since the protein levels of pro-inflammatory cytokines including IL-1α, IL-1β, IL-6 and TNF-α were reduced following DIZE treatment. Previously, we and others revealed that MAS1 was expressed by microglia and astrocytes, the main immune cells in the brain [18, 28, 29]. More importantly, mounting evidence suggested that Ang-(1-7) bound to MAS1 receptors and thus inhibited inflammatory responses in the brain under several pathological conditions including ischemic stroke and AD [29–31]. Since activation of ACE2 by DIZE led to elevated Ang-(1-7) levels and a higher expression of MAS1, Ang-(1-7)/MAS1-mediated signaling pathway might contribute to the anti-inflammatory effects of DIZE in this scenario.

In the current study, we showed that DIZE treatment provided neuroprotection in SAMP8 mice, since neuronal and synaptic losses in the brain were rescued by DIZE. Interestingly, the same protection was observed in a d-galactose-ovariectomized rat model of AD after DIZE treatment [14]. Meanwhile, the protective effects of DIZE on neuron and synapse were also noted in animal models of other neurological disorders, such as cerebral ischemia and multiple sclerosis [32, 33]. This beneficial effect seemed to be a consequence of the DIZE-induced amelioration of AD-like neuropathology. However, we cannot rule out a direct protection of neuron and synapse caused by DIZE, since DIZE was revealed to block acid-sensing ion channels and thus supported neuronal survival and restored synapse density in mice with experimental autoimmune encephalomyelitis [33].

More importantly, we demonstrated that DIZE treatment improved the performance of SAMP8 mice in MWM tests, indicating a protective effect of DIZE against AD-related spatial cognitive impairment. This finding was supported by a recent study showing that DIZE administration prevented the development of cognitive deficits in a transgenic AD animal model [15]. Since neuronal and synaptic integrity in these brain regions was required for the maintenance of normal cognitive functions [34], the improvement in spatial cognition following DIZE treatment could be a consequence of the attenuated synaptic and neuronal losses in the brain. It is worth noting that our results did not support a direct influence of DIZE on spatial cognitive functions, since the performance of SAMR1 control mice in MWM tests was not significantly improved by DIZE.

It should be noted that this study has some limitations. First, although SAMP8 mouse was considered as a sporadic AD animal model, many researchers argued that its pathological alterations were not equal to those during AD progression. Therefore, our findings should be further confirmed using classic transgenic AD animal models, such as *APP*/*PS1* mouse. Second, in the current study, we did not establish the causality between activation of brain ACE2/Ang-(1-7)/MAS1 axis and the DIZE-mediated neuroprotection. This potential causal relationship should be validated using ACE2 inhibitors or MAS1 receptor antagonists in the future. Third, in most of our experiments, tissue samples of the whole brain were used. This might mask potential region-specific alterations induced by DIZE. In our future studies, effects of DIZE on neuropathological changes in AD-related brain regions should be evaluated.

In conclusion, this study demonstrates that DIZE ameliorates AD-like neuropathology and rescues cognitive impairment in SAMP8 mice. Meanwhile, these beneficial effects of DIZE may be achieved by activating brain ACE2/Ang-(1-7)/MAS1 axis. These findings highlight brain ACE2/Ang-(1-7)/MAS1 axis as a potential target for the treatment of sporadic AD.

## MATERIALS AND METHODS

### Ethics statement

This study was reported in accordance with the ARRIVE guideline [35], and the procedures involving animals were in accordance with the ethical standards of Nanjing First Hospital (protocol#: IACUC-191032).

### Animals and treatments

To avoid the interference of estrogen on AD-like neuropathology and cognitive functions [36, 37], only male animals were used in this study. SAMP8 mice were established through phenotypic selection from a common genetic pool of AKR/J strain of mice in 1981 by Takeda and colleagues [38]. SAMP8 mice are commonly used as animal models of aging-related diseases such as sporadic AD while SAMR1 mice are often employed as their normal aging controls [16, 17]. Eight-month-old male SAMP8 mice and their age-matched SAMR1 control mice were purchased from Beijing HFK Bioscience Company. According to our previous findings, at this age, SAMP8 mice have developed AD-like neuropathology including overproduction of Aβ, tau hyperphosphorylation and neuroinflammation [39]. Mice were maintained in individually ventilated cages in a standard animal room with a 12 h light/dark cycle and given free access to food and water as described [18].

Mice were randomly allocated to each group using a random number table generated by SPSS 16.0 software (IBM, Armonk, NY, USA), and were injected intraperitoneally with vehicle (0.9% sterile saline) or DIZE (#HY-12404, MedChemExpress LLC, Monmouth Junction, NJ, USA; Prepared daily in 0.9% sterile saline) once a day for 30 consecutive days. Afterward, mice were sacrificed for analysis. The dose and route of DIZE administration were chosen according to previous studies [14, 15]. During the whole experiment, we carefully monitored the general health of mice and did not observe obvious adverse effects or significant changes in their body weight or food intakes. Systolic blood pressure (SBP) was measured at the beginning and the end of the treatment period using the tail-cuff method as described [40]. No significant difference was found in SBP between SAMP8 mice and their age-matched SAMR1 mice at the baseline (data not shown). Meanwhile, DIZE (5 mg/kg and 15 mg/kg) did not significantly affect SBP at the end of the treatment period (data not shown).

### Western blot analysis

Western blot was carried out as described [41]. The whole brain was lysed in an extraction buffer containing complete protease inhibitor cocktail. Different samples with an equal amount of protein were separated on SDS polyacrylamide gels, transferred to PVDF membranes, and then blocked with non-fat milk for 1 h. Membranes were then incubated overnight at 4°C with the primary antibody against tau hyperphosphorylated at Thr205 (1:1000, #SAB4504561, Sigma-Aldrich, Inc., St. Louis, MO, USA), tau hyperphosphorylated at Ser396 (1:1000, #44-752G, Thermo Fisher Scientific, Waltham, MA, USA), total tau protein (1:800, #SAB4501831, Sigma-Aldrich, Inc., St. Louis, MO, USA), MAS1 (1:1000, #ab235914, Abcam plc., Cambridge, MA, USA) or synaptophysin (1:1500, #SAB4502906, Sigma-Aldrich, Inc., St. Louis, MO, USA), then washed again and incubated with appropriate horseradish peroxidase (HRP)-coupled secondary antibody for another 2 h. After washing, protein bands were detected with chemiluminescent HRP substrate (#32132, Thermo Fisher Scientific, Waltham, MA, USA) for 5 min. The signal intensity of primary antibody binding was analyzed using Quantity One software (Bio-Rad Laboratories, Inc., Hercules, CA, USA). β-actin was used as a loading control (1:1500, #4970, Cell Signaling Technology, Inc., Danvers, MA, USA).

### ELISA

Tris-buffered saline (TBS)-soluble Aβ_1-42_ levels in the brain of mice were detected as described [42]. The whole brain of mouse was homogenized in 10 volumes of TBS containing 5 mM ethylene diamine tetraacetic acid (EDTA), phosphatase inhibitor, EDTA-free protease inhibitor cocktail and 2 mM 1,10-phenanthroline at 4 °C. The homogenate was centrifuged at 100,000g for 1 h at 4 °C. Supernatants were collected, and TBS-soluble Aβ_1-42_ was measured by a commercial ELISA kit (#KMB3441, Thermo Fisher Scientific, Waltham, MA, USA).

The protein levels of Ang-(1-7) and inflammatory cytokines including IL-1α, IL-1β, IL-6 and TNF-α in the whole brain of mice were measured by commercial ELISA kits (For Ang-(1-7): #S-1330; Bachem Inc., Torrance, CA, USA; For IL-1α: #MLA00, R&D Systems, Inc., Minneapolis, MN, USA; For IL-1β: #MLB00C, R&D Systems, Inc., Minneapolis, MN, USA; For IL-6: #M6000B, R&D Systems, Inc., Minneapolis, MN, USA; For TNF-α: #MTA00B, R&D Systems, Inc., Minneapolis, MN, USA) as described [18, 39].

### ACE2 activity measurement

ACE2 activity in the whole brain was detected using a commercial ACE2 activity assay kit (#AS-72086, AnaSpec, Inc., Fremont, CA, USA) with Mc-Ala/Dnp fluorescence resonance energy transfer peptides as described [7]. The fluorescence of Mc-Ala was monitored at excitation/emission 330 nm/390 nm. The specificity was confirmed using a specific ACE2 inhibitor DX600 (#AS-62337, AnaSpec, Inc., Fremont, CA, USA).

### qRT-PCR

Total RNA in the whole brain was extracted by Trizol reagent as described [18]. Equal amounts of total RNA were reverse transcribed using the PrimeScript™ RT Master Mix (Takara Bio, Inc., Kusatsu, Shiga, Japan) under standard conditions. Afterward, qRT-PCR reactions were performed with specific primers (*Mas1* forward: CATCTAGGACTGGGCAGAGC, *Mas1* reverse: AGTCAGGAGGTGGAGAGCAA. *Gapdh* forward: CAACAGCAACTCCCACTCTTC, *Gapdh* reverse: GGTCCAGGGTTTCTTACTCCTT) and SYBR Green Premix Ex Taq (Takara Bio, Inc., Kusatsu, Shiga, Japan).

### Nissl staining

Nissl staining was performed as described [39]. Briefly, the paraffin-embedded sections were dewaxed and rehydrated according to the standard protocols. Next, sections were stained in 1% cresyl violet at 50°C for 5 min. After being rinsed with water, sections were dehydrated in increasing concentrations of ethanol, mounted on the slides, and examined with a light microscope. Three coronal sections at different depths on the rostro-caudal axis were imaged for each animal, and six fields of cortex and hippocampus on each coronal section were randomly selected for quantitative analysis. Neurons with dark violet nucleus and intact morphology were identified as Nissl-positive neurons, and the numbers of Nissl-positive neurons were counted by observers who were unaware of the experimental groups.

### MWM test

MWM test has been performed during the last 5 days before mice were killed as described [39]. Mice were given four training trials per day for 5 consecutive days. The path length to the submerged platform was recorded by a video camera, and the average value of four trials was calculated.

### Statistical analysis

Data were analyzed using GraphPad Prism 8 (GraphPad Software, San Diego, CA, USA) as described [18]. One-way ANOVA followed by Tukey’s post hoc test was employed to analyze differences among groups. Data were expressed as mean ± SD. *P*<0.05 was considered statistically significant.

## References

[1] Scheltens P, Blennow K, Breteler MM, de Strooper B, Frisoni GB, Salloway S, Van der Flier WM. Alzheimer’s disease. Lancet. 2016; 388:505–17. 10.1016/S0140-6736(15)01124-1 26921134

[2] Lane CA, Hardy J, Schott JM. Alzheimer’s disease. Eur J Neurol. 2018; 25:59–70. 10.1111/ene.13439 28872215

[3] Jiang T, Yu JT, Tian Y, Tan L. Epidemiology and etiology of alzheimer’s disease: from genetic to non-genetic factors. Curr Alzheimer Res. 2013; 10:852–67. 10.2174/15672050113109990155 23919770

[4] Reitz C, Mayeux R. Alzheimer disease: epidemiology, diagnostic criteria, risk factors and biomarkers. Biochem Pharmacol. 2014; 88:640–51. 10.1016/j.bcp.2013.12.024 24398425 PMC3992261

[5] Xu P, Sriramula S, Lazartigues E. ACE2/ANG-(1-7)/mas pathway in the brain: the axis of good. Am J Physiol Regul Integr Comp Physiol. 2011; 300:R804–17. 10.1152/ajpregu.00222.2010 21178125 PMC3075080

[6] Jiang T, Tan L, Gao Q, Lu H, Zhu XC, Zhou JS, Zhang YD. Plasma angiotensin-(1-7) is a potential biomarker for alzheimer’s disease. Curr Neurovasc Res. 2016; 13:96–99. 10.2174/1567202613666160224124739 26907614

[7] Jiang T, Zhang YD, Zhou JS, Zhu XC, Tian YY, Zhao HD, Lu H, Gao Q, Tan L, Yu JT. Angiotensin-(1-7) is reduced and inversely correlates with tau hyperphosphorylation in animal models of alzheimer’s disease. Mol Neurobiol. 2016; 53:2489–97. 10.1007/s12035-015-9260-9 26044748

[8] Marshall AC, Shaltout HA, Pirro NT, Rose JC, Diz DI, Chappell MC. Enhanced activity of an angiotensin-(1-7) neuropeptidase in glucocorticoid-induced fetal programming. Peptides. 2014; 52:74–81. 10.1016/j.peptides.2013.12.006 24355101 PMC4157337

[9] Domenig O, Manzel A, Grobe N, Kaltenecker C, Kovarik J, Stegbauer J, Gurley SB, Antlanger M, Elased K, Saemann M, Linker R, Poglitsch M. 8D.05: The Role of Neprilysin in Angiotensin 1-7 Formation in the Kidney. J Hypertens. 2015; 33:e114–e115. 10.1097/01.hjh.0000467659.31038.0f

[10] Vickers C, Hales P, Kaushik V, Dick L, Gavin J, Tang J, Godbout K, Parsons T, Baronas E, Hsieh F, Acton S, Patane M, Nichols A, Tummino P. Hydrolysis of biological peptides by human angiotensin-converting enzyme-related carboxypeptidase. J Biol Chem. 2002; 277:14838–43. 10.1074/jbc.M200581200 11815627

[11] Elased KM, Cunha TS, Marcondes FK, Morris M. Brain angiotensin-converting enzymes: role of angiotensin-converting enzyme 2 in processing angiotensin II in mice. Exp Physiol. 2008; 93:665–75. 10.1113/expphysiol.2007.040311 18263657 PMC7197900

[12] Bennion DM, Haltigan EA, Irwin AJ, Donnangelo LL, Regenhardt RW, Pioquinto DJ, Purich DL, Sumners C. Activation of the neuroprotective angiotensin-converting enzyme 2 in rat ischemic stroke. Hypertension. 2015; 66:141–48. 10.1161/HYPERTENSIONAHA.115.05185 25941346 PMC4465873

[13] Wang L, de Kloet AD, Pati D, Hiller H, Smith JA, Pioquinto DJ, Ludin JA, Oh SP, Katovich MJ, Frazier CJ, Raizada MK, Krause EG. Increasing brain angiotensin converting enzyme 2 activity decreases anxiety-like behavior in male mice by activating central mas receptors. Neuropharmacology. 2016; 105:114–23. 10.1016/j.neuropharm.2015.12.026 26767952 PMC4873386

[14] Kamel AS, Abdelkader NF, Abd El-Rahman SS, Emara M, Zaki HF, Khattab MM. Stimulation of ACE2/ANG(1-7)/mas axis by diminazene ameliorates alzheimer’s disease in the d-galactose-ovariectomized rat model: role of PI3K/Akt pathway. Mol Neurobiol. 2018; 55:8188–202. 10.1007/s12035-018-0966-3 29516284

[15] Evans CE, Miners JS, Piva G, Willis CL, Heard DM, Kidd EJ, Good MA, Kehoe PG. ACE2 activation protects against cognitive decline and reduces amyloid pathology in the Tg2576 mouse model of alzheimer’s disease. Acta Neuropathol. 2020; 139:485–502. 10.1007/s00401-019-02098-6 31982938 PMC7035243

[16] Butterfield DA, Poon HF. The senescence-accelerated prone mouse (SAMP8): a model of age-related cognitive decline with relevance to alterations of the gene expression and protein abnormalities in alzheimer’s disease. Exp Gerontol. 2005; 40:774–83. 10.1016/j.exger.2005.05.007 16026957

[17] Morley JE, Farr SA, Kumar VB, Armbrecht HJ. The SAMP8 mouse: a model to develop therapeutic interventions for alzheimer’s disease. Curr Pharm Des. 2012; 18:1123–30. 10.2174/138161212799315795 22288401

[18] Jiang T, Xue LJ, Yang Y, Wang QG, Xue X, Ou Z, Gao Q, Shi JQ, Wu L, Zhang YD. AVE0991, a nonpeptide analogue of ang-(1-7), attenuates aging-related neuroinflammation. Aging (Albany NY). 2018; 10:645–57. 10.18632/aging.101419 29667931 PMC5940107

[19] Xie W, Zhu D, Ji L, Tian M, Xu C, Shi J. Angiotensin-(1-7) improves cognitive function in rats with chronic cerebral hypoperfusion. Brain Res. 2014; 1573:44–53. 10.1016/j.brainres.2014.05.019 24854124

[20] Hardy J. Alzheimer’s disease: the amyloid cascade hypothesis: an update and reappraisal. J Alzheimers Dis. 2006; 9:151–53. 10.3233/jad-2006-9s317 16914853

[21] Crackower MA, Sarao R, Oudit GY, Yagil C, Kozieradzki I, Scanga SE, Oliveira-dos-Santos AJ, da Costa J, Zhang L, Pei Y, Scholey J, Ferrario CM, Manoukian AS, et al. Angiotensin-converting enzyme 2 is an essential regulator of heart function. Nature. 2002; 417:822–28. 10.1038/nature00786 12075344

[22] Liu S, Liu J, Miura Y, Tanabe C, Maeda T, Terayama Y, Turner AJ, Zou K, Komano H. Conversion of Aβ43 to Aβ40 by the successive action of angiotensin-converting enzyme 2 and angiotensin-converting enzyme. J Neurosci Res. 2014; 92:1178–86. 10.1002/jnr.23404 24823497

[23] Bloom GS. Amyloid-β and tau: the trigger and bullet in alzheimer disease pathogenesis. JAMA Neurol. 2014; 71:505–08. 10.1001/jamaneurol.2013.5847 24493463 PMC12908160

[24] Su Z, Zimpelmann J, Burns KD. Angiotensin-(1-7) inhibits angiotensin II-stimulated phosphorylation of MAP kinases in proximal tubular cells. Kidney Int. 2006; 69:2212–18. 10.1038/sj.ki.5001509 16672906

[25] Munoz L, Ammit AJ. Targeting p38 MAPK pathway for the treatment of alzheimer’s disease. Neuropharmacology. 2010; 58:561–68. 10.1016/j.neuropharm.2009.11.010 19951717

[26] Maphis N, Jiang S, Xu G, Kokiko-Cochran ON, Roy SM, Van Eldik LJ, Watterson DM, Lamb BT, Bhaskar K. Selective suppression of the α isoform of p38 MAPK rescues late-stage tau pathology. Alzheimers Res Ther. 2016; 8:54. 10.1186/s13195-016-0221-y 27974048 PMC5157054

[27] Hensley K. Neuroinflammation in alzheimer’s disease: mechanisms, pathologic consequences, and potential for therapeutic manipulation. J Alzheimers Dis. 2010; 21:1–14. 10.3233/JAD-2010-1414 20182045 PMC3792565

[28] Moore ED, Kooshki M, Metheny-Barlow LJ, Gallagher PE, Robbins ME. Angiotensin-(1-7) prevents radiation-induced inflammation in rat primary astrocytes through regulation of MAP kinase signaling. Free Radic Biol Med. 2013; 65:1060–68. 10.1016/j.freeradbiomed.2013.08.183 24012919 PMC3879043

[29] Liu M, Shi P, Sumners C. Direct anti-inflammatory effects of angiotensin-(1-7) on microglia. J Neurochem. 2016; 136:163–71. 10.1111/jnc.13386 26448556 PMC4688174

[30] Chen JL, Zhang DL, Sun Y, Zhao YX, Zhao KX, Pu D, Xiao Q. Angiotensin-(1-7) administration attenuates alzheimer’s disease-like neuropathology in rats with streptozotocin-induced diabetes via mas receptor activation. Neuroscience. 2017; 346:267–77. 10.1016/j.neuroscience.2017.01.027 28147245

[31] Jiang T, Gao L, Guo J, Lu J, Wang Y, Zhang Y. Suppressing inflammation by inhibiting the NF-κB pathway contributes to the neuroprotective effect of angiotensin-(1-7) in rats with permanent cerebral ischaemia. Br J Pharmacol. 2012; 167:1520–32. 10.1111/j.1476-5381.2012.02105.x 22817481 PMC3514764

[32] Mecca AP, Regenhardt RW, O’Connor TE, Joseph JP, Raizada MK, Katovich MJ, Sumners C. Cerebroprotection by angiotensin-(1-7) in endothelin-1-induced ischaemic stroke. Exp Physiol. 2011; 96:1084–96. 10.1113/expphysiol.2011.058578 21685445 PMC3210510

[33] de Ceglia R, Chaabane L, Biffi E, Bergamaschi A, Ferrigno G, Amadio S, Del Carro U, Mazzocchi N, Comi G, Bianchi V, Taverna S, Forti L, D’Adamo P, et al. Down-sizing of neuronal network activity and density of presynaptic terminals by pathological acidosis are efficiently prevented by diminazene aceturate. Brain Behav Immun. 2015; 45:263–76. 10.1016/j.bbi.2014.12.003 25499583

[34] Walsh DM, Selkoe DJ. Deciphering the molecular basis of memory failure in alzheimer’s disease. Neuron. 2004; 44:181–93. 10.1016/j.neuron.2004.09.010 15450169

[35] Hooijmans CR, de Vries R, Leenaars M, Curfs J, Ritskes-Hoitinga M. Improving planning, design, reporting and scientific quality of animal experiments by using the gold standard publication checklist, in addition to the ARRIVE guidelines. Br J Pharmacol. 2011; 162:1259–60. 10.1111/j.1476-5381.2010.01128.x 21091655 PMC3058159

[36] Hamson DK, Roes MM, Galea LA. Sex hormones and cognition: neuroendocrine influences on memory and learning. Compr Physiol. 2016; 6:1295–337. 10.1002/cphy.c150031 27347894

[37] Zhao L, Woody SK, Chhibber A. Estrogen receptor β in alzheimer’s disease: from mechanisms to therapeutics. Ageing Res Rev. 2015; 24:178–90. 10.1016/j.arr.2015.08.001 26307455 PMC4661108

[38] Takeda T, Hosokawa M, Takeshita S, Irino M, Higuchi K, Matsushita T, Tomita Y, Yasuhira K, Hamamoto H, Shimizu K, Ishii M, Yamamuro T. A new murine model of accelerated senescence. Mech Ageing Dev. 1981; 17:183–94. 10.1016/0047-6374(81)90084-1 7311623

[39] Jiang T, Yu JT, Zhu XC, Tan MS, Gu LZ, Zhang YD, Tan L. Triggering receptor expressed on myeloid cells 2 knockdown exacerbates aging-related neuroinflammation and cognitive deficiency in senescence-accelerated mouse prone 8 mice. Neurobiol Aging. 2014; 35:1243–51. 10.1016/j.neurobiolaging.2013.11.026 24368090

[40] Jiang T, Gao L, Shi J, Lu J, Wang Y, Zhang Y. Angiotensin-(1-7) modulates renin-angiotensin system associated with reducing oxidative stress and attenuating neuronal apoptosis in the brain of hypertensive rats. Pharmacol Res. 2013; 67:84–93. 10.1016/j.phrs.2012.10.014 23127917

[41] Jiang T, Zhang YD, Gao Q, Ou Z, Gong PY, Shi JQ, Wu L, Zhou JS. TREM2 ameliorates neuronal tau pathology through suppression of microglial inflammatory response. Inflammation. 2018; 41:811–23. 10.1007/s10753-018-0735-5 29362997

[42] Jiang T, Zhang YD, Gao Q, Zhou JS, Zhu XC, Lu H, Shi JQ, Tan L, Chen Q, Yu JT. TREM1 facilitates microglial phagocytosis of amyloid beta. Acta Neuropathol. 2016; 132:667–83. 10.1007/s00401-016-1622-5 27670763

